# The effect of age and disease duration on the efficacy of subthalamic nuclei deep brain stimulation in Parkinson's disease patients

**DOI:** 10.1111/cns.13958

**Published:** 2022-09-07

**Authors:** Tingting Du, Tianshuo Yuan, Guanyu Zhu, Ruoyu Ma, Xin Zhang, Yingchuan Chen, Jianguo Zhang

**Affiliations:** ^1^ Department of Functional Neurosurgery Beijing Neurosurgical Institute, Capital Medical University Beijing China; ^2^ Department of Neurosurgery, Beijing Tiantan Hospital Capital Medical University Beijing China; ^3^ Beijing Key Laboratory of Neurostimulation Beijing China

**Keywords:** age, disease duration, Parkinson's disease, subthalamic nucleus deep brain stimulation, therapeutic efficacy

## Abstract

**Background:**

Previous studies have reported the effects of age and disease duration on the efficacy of subthalamic nuclei deep brain stimulation (STN‐DBS) of Parkinson's disease (PD) patients. However, available data involving these issues are not consistent. In particular, the effect of age and disease duration on the initial efficacy of STN‐DBS has not been established.

**Methods:**

A total of 51 patients with PD treated with bilateral STN‐DBS were involved in the present study. They received clinical symptom evaluation during the preoperative, initial, and chronic stages of surgery. The correlations between age when undergoing surgery/age at disease onset/disease duration and outcomes of STN‐DBS were measured.

**Results:**

The preoperative levodopa response was negatively associated with age. During the initial stage, the age when undergoing surgery and age at disease onset were negatively correlated with the effect on bradykinesia, with better symptom control of general symptoms in long‐term disease patients. Similarly, patients with an early time of surgery and disease onset and long‐term disease duration showed better control of bradykinesia and axial symptoms at the chronic stage. Furthermore, a long‐term disease duration and early disease onset benefited from an increase of therapeutic efficacy in general, rigid, and axial symptoms with STN‐DBS after a long period. Nevertheless, patients with late disease onset achieved a better relief of stigma.

**Conclusion:**

Age and disease durations played a unique role in controlling the symptoms of PD patients treated with STN‐DBS. These results may contribute to patient selection and adjustments of expectations of surgery, based on the age, disease duration, and different symptoms.

## Graphical abstract

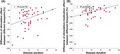

## 1 INTRODUCTION

Parkinson disease (PD) is one of the most common neurodegenerative diseases and is clinically characterized by a series of motor impairments, including tremor, rigidity, and bradykinesia. PD is also associated with non‐motor symptoms, such as cognitive and emotional impairments.^1^ Furthermore, subthalamic nuclei deep brain stimulation (STN‐DBS) is a well‐established therapy for relieving these symptoms of PD.^2^, ^3^

PD prevalence is increasing with age, with PD affecting 1% of the population greater than 60 years of age; however, more than 4% of the population greater than 85 years of age suffers from PD.^4^ Compared with early‐onset PD patients, significantly more severe motor impairments are observed in patients with a later onset but with similar disease durations.^5^ A previous study showed that there was a significant negative correlation between age and the levodopa response.^6^ Similarly, age has a great impact on the outcomes of STN‐DBS when treating PD patients. After chronic stimulation, there is a significant negative correlation between age and the improvement of some parameters of health‐related quality.^7^

STN‐DBS is typically used after the disease has been present for 11–13 years; however, recent studies reported that STN‐DBS treatment was superior to medical therapy in patients with early PD, indicating that young patients and patients with short disease duration may undergo this surgery.^8^, ^9^ Some recent studies also found that elderly patients undergoing DBS did not have an increased risk of more serious complications.^10^ However, the results of these studies were not consistent. To the best of our knowledge, no study has investigated the effects of age and disease duration on the efficacy of initial STN‐DBS treatment of PD patients; therefore, these effects were investigated in the present study.

## 2 MATERIALS AND METHODS

### 2.1 Patients and the evolution of clinical symptoms

A total of 51 patients with PD treated with bilateral STN‐DBS at Beijing Tiantan Hospital between 2015 and 2019 were retrospectively studied. The inclusion criteria were as follows: (1) a diagnosis of PD based on the UK brain bank criteria^11^; (2) patients treated with bilateral STN‐DBS; and (3) patients receiving clinical assessments at different time points, including the preoperative, initial (1 month post‐operation), and chronic (12 months post‐operation) stages. The exclusion criteria included (1) having a history of prior thalamotomy or other cerebral neurosurgery and (2) an incomplete scale evaluation.

Clinical variables, including sex, age, disease duration before surgery, Hoehn‐Yahr (H‐Y) stage, and levodopa equivalent dose (LEDD, based on a previous study^12^), were recorded. Clinical symptom evolution was assessed using the Unified Parkinson Disease Rating Scale III (UPDRS III, evaluating motor impairment), the 39‐item Parkinson's disease questionnaire (PDQ‐39, evaluating health‐related quality), freezing of gait questionnaire (FOGQ, evaluating gait freezing), the Montreal cognitive assessment (MoCA, evaluating cognitive impairment), the Hamilton anxiety rating scale (HAMA, evaluating anxiety), and the Hamilton depression rating scale (HDRS, evaluating depression). The UPDRS III was divided into different subscores of tremor (UPDRS items 20, 21; range: 0–28); rigidity (UPDRS item 22; scores: 0–20); bradykinesia (UPDRS items 23, 24, 25, 26, and 31; range: 0–36); and axial symptoms (UPDRS items 18, 27, 28, 29, and 30; range: 0–20).

### 2.2 Surgical procedures

Bilateral STN‐DBS implantations were conducted using a Leksell G frame system with the assistance of a Leksel Surgiplan workstation (Elekta Instrument AB, Stockholm, Sweden) with preoperative magnetic resonance imaging and computed tomography. Micro‐electrode recordings and macro‐stimulation were used to accurately target the STN. During electrode implantation, the steel cannulas were kept in place. Quadripolar DBS electrodes (Model 3389; Medtronic; Model L301; PINS Medical; using the same parameters) were implanted and fixed, and the implanted pulse generator was then implanted.

After 4–5 weeks (1 month post‐operation), the patients were asked to return to the hospital to start the program (in a stable off medication condition), to minimize micro‐subthalamotomy effects.^13^ Each patient underwent a regular adjustment of stimulation settings, achieving satisfactory clinical outcomes and avoiding intolerable side effects. The clinical symptoms were measured, and then each patient underwent a regular adjustment of stimulation settings and medications until optimal control of symptoms was established, followed by a chronic stage clinical evaluation (12 ± 1 months post‐operation).

### 2.3 Total electrical energy delivered (TEED) calculation

TEED, the total energy delivered by the DBS system over a specific period of time, was determined using the programmed parameters of stimulation and the measured system impedance, which was developed by a previous study and used to evaluate the stimulation intensity.^14^ The formula was as follows: TEED (1 s) = [(voltage^2^ × pulse width × frequency)/impedance].

### 2.4 Statistical analysis

All data are expressed as the mean ± standard deviation or as a median (Q1, Q3). The influence of age when undergoing surgery, age at disease onset, and disease duration on the efficacy of STN‐DBS (and symptoms of PD and efficacies of medications) were investigated using Pearson's correlations between age when undergoing surgery/age at disease onset/disease duration and the efficacy/symptom severity, for each continuous variable. The changes of symptoms at different time points were measured using paired *t*‐test or Wilcoxon signed‐rank test. All statistical analyses were conducted using MATLAB (2019b; Mathworks). The medication, stimulation and optimal (stimulation + medication) effects on UPDRS III were calculated as follows: $\text{Medication response}\;\left(\text{rate}\right)=\frac{{\text{UPDRS}}_{\mathrm{pre}:\mathrm{med}-\mathrm{off}}-{\text{UPDRS}}_{\mathrm{pre}:\mathrm{med}-\mathrm{on}}}{{\text{UPDRS}}_{\mathrm{pre}:\mathrm{med}-\mathrm{off}}}\times 100\%$, $\text{Stimulation effect}\;\left(\text{rate}\right)=\frac{{\text{UPDRS}}_{\mathrm{pre}:\mathrm{med}-\mathrm{off}}-{\text{UPDRS}}_{\text{post}:\mathrm{stm}-\mathrm{on}\&\mathrm{med}-\mathrm{off}}}{{\text{UPDRS}}_{\mathrm{pre}:\mathrm{med}-\mathrm{off}}}\times 100\%$, and $\text{Optimal effect}\;\left(\text{rate}\right)=\frac{{\text{UPDRS}}_{\mathrm{pre}:\mathrm{med}-\mathrm{off}}-{\text{UPDRS}}_{\text{post}:\mathrm{stm}-\mathrm{on}\&\mathrm{med}-\mathrm{on}}}{{\text{UPDRS}}_{\mathrm{pre}:\mathrm{med}-\mathrm{off}}}\times 100\%$.

The therapeutic outcome differences between the initial and chronic stages were further measured using the following:

$\text{Stimulation effect change}\;\left(\text{rate}\right)=\frac{{\text{UPDRS}}_{1\;\text{month}:\mathrm{stm}-\mathrm{on}\&\mathrm{med}-\mathrm{off}}-{\text{UPDRS}}_{12\;\text{month}:\mathrm{stm}-\mathrm{on}\&\mathrm{med}-\mathrm{off}}}{{\text{UPDRS}}_{1\;\text{month}:\mathrm{stm}-\mathrm{on}\&\mathrm{med}-\mathrm{off}}}\times 100\%$.

$\text{Optimal effect change}\;\left(\text{rate}\right)=\frac{{\text{UPDRS}}_{1\;\text{month}:\mathrm{stm}-\mathrm{on}\&\mathrm{med}-\mathrm{on}}-{\text{UPDRS}}_{12\;\text{month}:\mathrm{stm}-\mathrm{on}\&\mathrm{med}-\mathrm{on}}}{{\text{UPDRS}}_{1\;\text{month}:\mathrm{stm}-\mathrm{on}\&\mathrm{med}-\mathrm{on}}}\times 100\%$.

The subscores of UPDRS III, including tremor, rigidity, bradykinesia, and axial symptoms, were only measured for changes in values, instead of the rates, because some subscores were zero, which could not be used as a denominator. Other scales, such as PDQ‐39, HAMA, and HDRS were measured in a similar manner. A value of *p* < 0.05 was considered to indicate statistical significance.

## 3 RESULTS

### 3.1 Patient demographics

A total of 51 PD patients (30 males and 21 females) with bilateral STN‐DBS were involved in the present study. The mean age when undergoing surgery was 62.47 ± 7.73 years. The mean age at disease onset was 52.23 ± 9.07 years. The mean disease duration was 10.24 ± 4.31 years, and the median H‐Y stage was 3.0 (3.0, 3.0).

The patients showed significant motor improvements after being treated with medications (*p* < 0.0001). Importantly, the initial and chronic optimal therapeutic efficacies (stimulation + medication) were both better than preoperative medication efficacies (initial: *p* < 0.0001; chronic: *p* = 0.0002); however, there was no significant difference of motor impairment between the initial and chronic stages (medication on: *p* = 0.3610; medication off: *p* = 0.3103). Regarding different symptoms of PD, including tremor, rigidity, bradykinesia, and axial sub‐scores, patients showed therapeutic outcomes of previous medications (all, *p* < 0.0001). The initial and chronic optimal therapeutic efficacies of all four motor subscores were significantly better than after a single medication (initial stage vs. preoperative: tremor, *p* = 0.0009; rigidity, *p* = 0.0001; bradykinesia, *p* = 0.0004; axial, *p* < 0.0001) (chronic stage vs. preoperative: tremor, *p* = 0.0002; rigidity, *p* < 0.0001; bradykinesia, *p* = 0.0002; and axial, *p* = 0.0066). However, there was no significant difference of motor impairment between the initial and chronic stages (medication on: tremor, *p* = 0.2081; rigidity, *p* = 0.1059; bradykinesia, *p* = 0.4674; axial, *p* = 0.1359) (medication off: tremor, *p* = 0.0984; rigidity, *p* = 0.2753; bradykinesia, *p* = 0.4595; axial, *p* = 0.6573). The severity of freezing of gait (FOG) was also significantly reduced with STN‐DBS (*p* < 0.0001), as was the LEDD (*p* < 0.0001) (Table 1).

**TABLE 1:** Clinical evaluation at different time points

	Pre‐operation	1‐month post‐operation	12‐month post‐operation
UPDRS III (med on/ med off)	24.27 ± 13.58/47.55 ± 15.61	15.65 ± 8.24/25.80 ± 11.32	14.53 ± 7.07/24.10 ± 11.05
Tremor	3.67 ± 3.99/8.43 ± 5.50	1.49 ± 2.14/3.08 ± 3.17	1.12 ± 2.04/2.45 ± 3.75
Rigidity	4.35 ± 3.72/9.65 ± 4.17	2.25 ± 2.53/5.02 ± 4.11	1.57 ± 1.68/4.16 ± 2.72
Bradykinesia	10.39 ± 5.47/18.96 ± 6.40	7.57 ± 4.38/11.43 ± 5.38	7.08 ± 3.98/10.90 ± 5.42
Axial	4.45 ± 2.43/8.47 ± 3.55	3.14 ± 2.03/4.78 ± 2.48	3.59 ± 2.18/5.00 ± 2.66
FOGQ	15.39 ± 7.36	–	9.39 ± 8.06
PDQ‐39	55.27 ± 21.94	–	36.29 ± 20.07
Mobility	18.31 ± 8.60	–	11.33 ± 8.81
Activities of daily life	11.41 ± 5.13	–	4.88 ± 3.97
Emotion of well‐being	7.25 ± 5.66	–	5.10 ± 4.94
Stigma	6.02 ± 4.88	–	3.78 ± 3.78
Social support	0.98 ± 1.74	–	1.27 ± 2.12
Cognition	4.98 ± 3.02	–	3.98 ± 2.96
Communication	3.25 ± 2.15	–	3.35 ± 2.42
Body discomfort	3.06 ± 2.53	–	2.59 ± 2.05
Summary index	31.64 ± 12.85	–	22.32 ± 11.91
MoCA	20.47 ± 4.95	–	22.75 ± 5.07
HAMA	15.14 ± 8.75	–	10.73 ± 7.34
HRSD	15.94 ± 7.76	–	10.78 ± 7.48
LEDD (mg/day)	756.48 ± 366.41	–	490.72 ± 230.50
TEED	–	–	107.51 ± 64.39

Abbreviations: FOGQ, freezing of gait questionnaire; HAMA, Hamilton anxiety rating scale; HDRS, Hamilton depression rating scale; LEDD, levodopa equivalent dose; MoCA, Montreal cognitive assessment; PDQ‐39, Parkinson's disease questionnaire‐39 items; TEED, total electrical energy delivered; UPDRS, Unified Parkinson Disease Rating Scale.

Similar with changes in motor performance, health‐related quality (PDQ‐39), cognitive impairment (MoCA), anxiety (HAMA), and depression (HDRS) symptoms were also decreased using STN‐DBS (PDQ‐39 and MoCA: all, *p* < 0.0001; HAMA: *p* = 0.0017; HDRS: *p* = 0.0002).

### 3.2 The effect of age and disease duration on motor impairments

**FIGURE 1:**
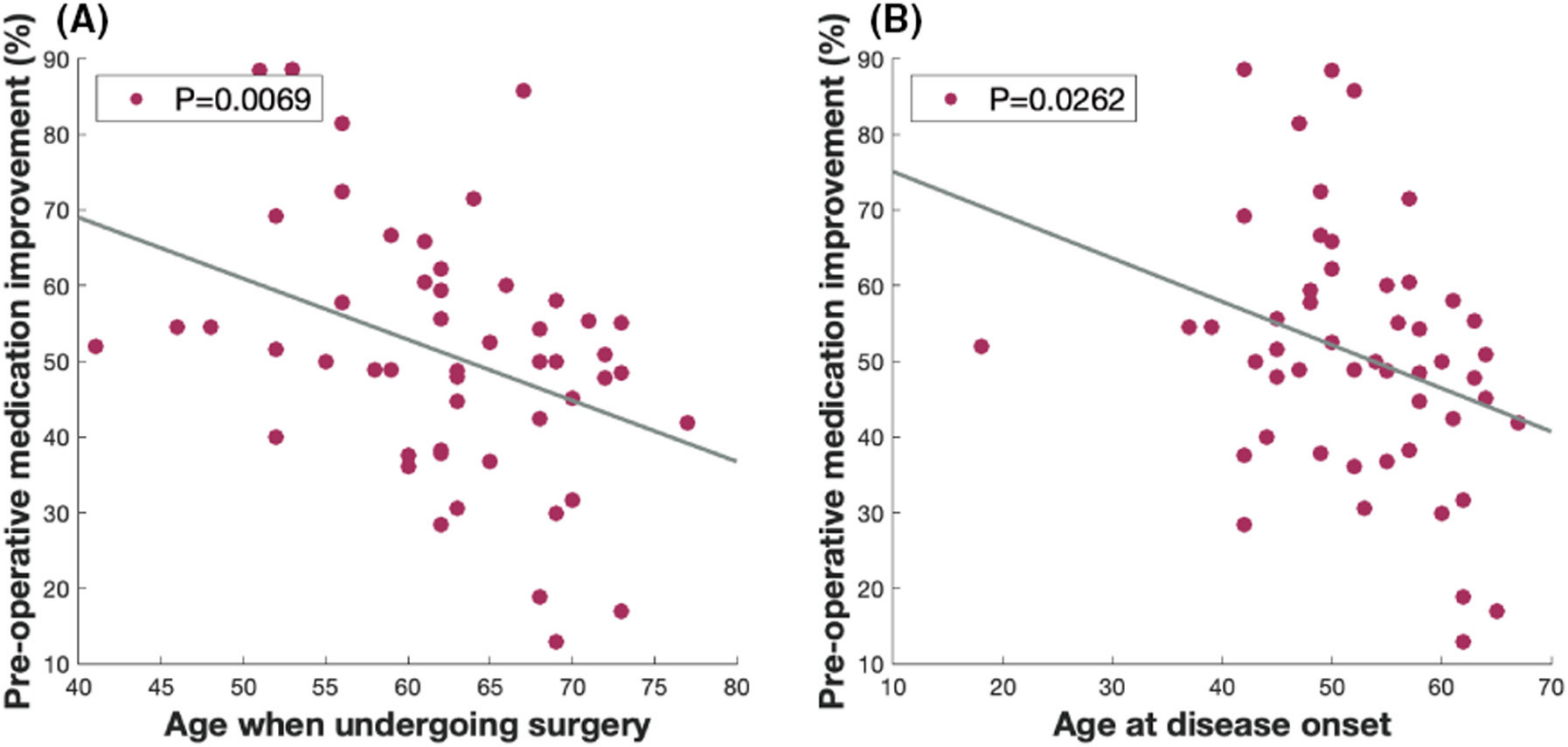
The relationship between medication response and age. The preoperative medication response (total Unified Parkinson Disease Rating Scale III score) was negatively correlated with the age when undergoing surgery (A) and age at disease onset (B)

Similar with a previous study,^6^ there was a negative correlation between age when undergoing surgery and preoperative medication response. This association might have been mainly attributed to bradykinesia and axial subscores of UPDRS III. Similar results were obtained between age at disease onset and preoperative medication response (Figure 1) (general, bradykinesia, and axial) (Table 2).

**TABLE 2:** The effect of age and disease duration on the motor efficacy of subthalamic nuclei deep brain stimulation

	Age underwent surgery(*p* value [+/−])	Age at disease onset (*p* value [+/−])	Disease duration (*p* value [+/−])
Pre‐operation			
Medication off‐bradykinesia	0.0381(−)	0.0405(−)	NS
Medication improvement‐total	0.0069(−)	0.0262(−)	NS
Medication improvement‐bradykinesia	0.0386(−)	0.0323(−)	NS
Medication improvement‐axial	0.0002(−)	0.0170(−)	NS
1‐month post‐operation			
Medication off‐total	NS	NS	0.0406(+)
Medication off‐rigidity	0.0403(−)	0.0121(−)	NS
Medication off‐axial	NS	NS	0.0056(+)
Stimulation effect‐rigidity (value)	NS	0.0173(+)	NS
Stimulation effect‐bradykinesia (value)	0.0330(−)	NS	NS
Stimulation+medication effect‐bradykinesia (value)	0.0097(−)	0.0251(−)	NS
Difference between medication and stimulation + medication‐total (rate, medication‐stimulation)	NS	NS	0.0229(+)
12‐month post‐operation			
Medication on‐axial	0.0035(+)	0.0048(+)	NS
Stimulation effect‐total (value)	NS	NS	NS
Stimulation effect‐bradykinesia (value)	0.0400(−)	0.0272(−)	NS
Stimulation effect‐axial (value)	0.0036(−)	0.0004(−)	0.0432(+)
Stimulation+medication effect‐bradykinesia (value)	0.0272(−)	0.0358(−)	NS
Stimulation+medication effect‐axial (value)	0.0060(−)	0.0132(−)	NS
Difference between medication and stimulation+medication‐axial (value, medication‐stimulation)	NS	NS	0.0196(+)
1 vs 12 months post‐operation (1–12 month)			
Stimulation+medication effect‐total (rate)	NS	NS	0.0229(+)
Stimulation+medication effect‐axial (value)	NS	0.0182(−)	0.0241(+)
Stimulation effect‐total (rate)	NS	NS	0.0175(+)
Stimulation effect‐total (value)	NS	0.0220(−)	0.0105(+)
Stimulation effect‐rigidity (value)	NS	0.0093(−)	0.0347(+)
Stimulation effect‐axial (value)	NS	0.0045(−)	0.0018(+)

Abbreviations: NS, not significant, *P* > 0.05; +, positive; −, negative.

For the initial stage of STN‐DBS, the stimulation effect on rigidity was positively associated with age at disease onset, whereas the stimulation effect on bradykinesia was negatively associated with the age when undergoing surgery. The optimal therapeutic efficacy (stimulation + medication) on bradykinesia was negatively correlated with age when undergoing surgery and age at disease onset. Moreover, the changes in optimal therapeutic efficacy (at the initial stage) and medication efficacy (preoperative) were positively associated with disease duration (Table 2).

For chronic stages of STN‐DBS, the age at disease onset was associated with the stimulation effect. Furthermore, stimulation and optimal therapeutic efficacy on bradykinesia and axial symptoms was both negatively correlated with age when undergoing surgery and age at disease onset. Notably, the disease duration was positively associated with the stimulation effect on axial symptoms and change in optimal therapeutic efficacies (axial symptoms during the chronic stage) and medication efficacies.

**FIGURE 2:**
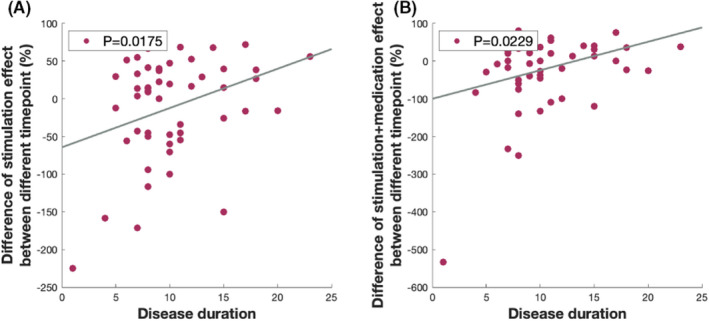
The relationship between alleviation of motor impairment and disease duration. The disease duration had a positive effect on elevation of stimulation (A) and optimal (B) effect on motor impairment between the initial and chronic stages

We further investigated the difference between the initial and chronic stages. The disease duration was positively associated with the stronger optimal therapeutic efficacies of general and axial symptoms, and the stimulation effects of general, rigid, and axial symptoms. The age at disease onset had a negative impact on stronger optimal therapeutic efficacies for axial symptoms, and stimulation effects on general, rigid, and axial symptoms (Figure 2, Table 2).

Up to 50% of patients with PD experience sudden and transient motor blocks (freezing) while initiating or performing activities.^15^ Giladi et al.^16^ developed the FOGQ, which is a clinician/interview administered patient‐reported rating scale, with higher scores denoting more severe FOG symptoms. However, we did not find a significant correlation between age/disease duration and the severity of FOG.

### 3.3 The effect of age and disease duration on health‐related quality

**FIGURE 3:**
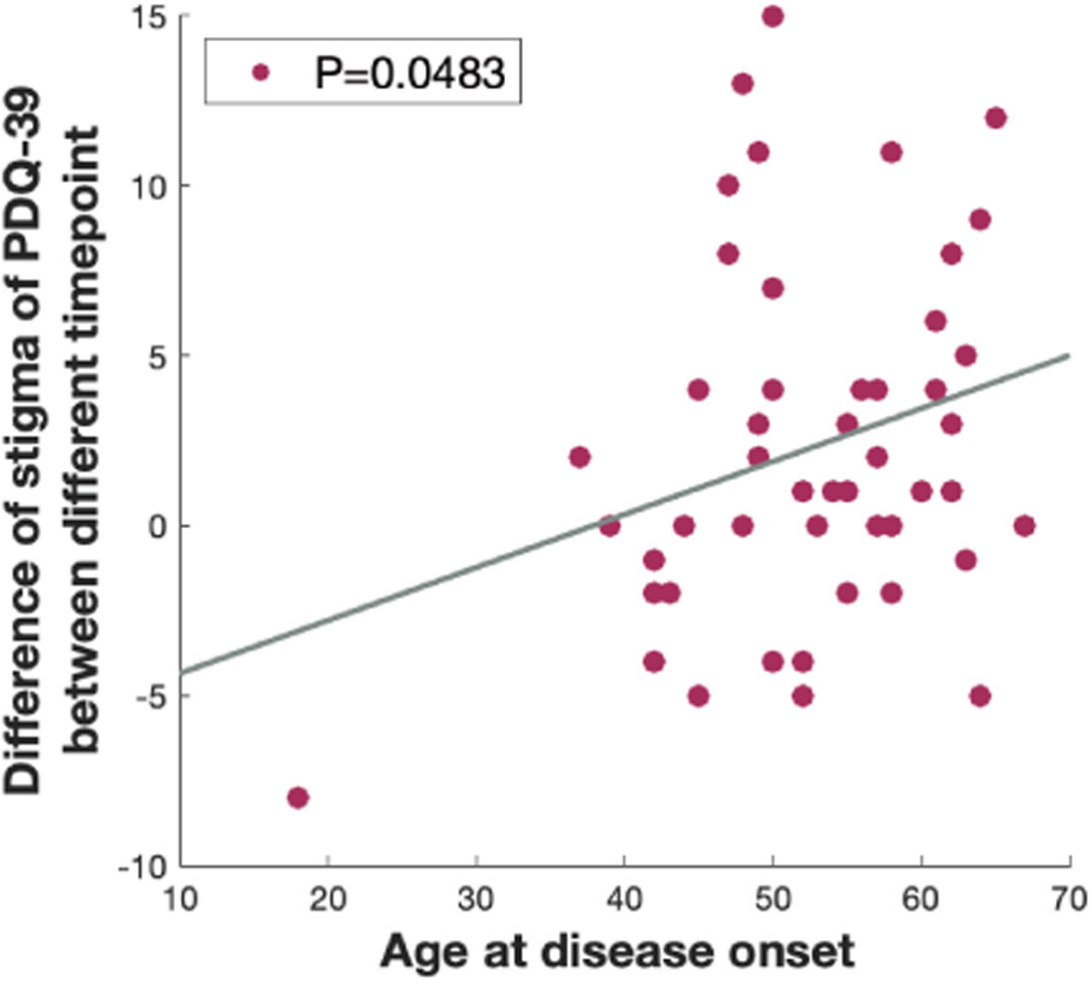
The relationship between age and stigma symptom. Older patients showed a better amelioration of stigma

PDQ‐39 assessment of PD‐specific health‐related quality over the last month could be divided into eight dimensions, involving mobility, activities of daily life, a feeling of well‐being, stigma, social support, cognition, communication, and bodily discomfort. The age when undergoing surgery and age at disease onset were negatively correlated with the impairment of emotions of well‐being and stigma dimensions during the chronic stage, whereas the age at disease onset was negatively associated with the severity of stigma and positively correlated with the severity of cognition during the chronic stage. Furthermore, we found that older patients showed a better amelioration of stigma (Figure 3, Table 3).

**TABLE 3:** The effect of age and disease duration on health‐related quality, cognition, and emotional efficacy of subthalamic nuclei deep brain stimulation

	Age underwent surgery (*p* value [+/−])	Age at disease onset (*p* value [+/−])	Disease duration (*p* value [+/−])
PDQ‐39			
12‐month post‐operation			
Emotion of well‐being	0.0260(−)	NS	NS
Stigma	0.0355(−)	0.0079(−)	NS
Cognition	NS	0.0250(+)	NS
Pre‐operation vs 12‐month post‐operation			
Stigma	NS	0.0483(+)	NS
MoCA			
Pre‐operation	0.0453(−)	0.0212(−)	NS
12‐month post‐operation	0.0059(−)	0.0069(−)	NS
HAMA			
12‐month post‐operation	NS	NS	0.0484(−)

Abbreviations: NS, not significant, *p* > 0.05; +, positive; −, negative.

### 3.4 The effect of age and disease duration on cognition and emotion

MoCA was used to evaluate cognitive impairments in PD patients, with a low score indicating a worse cognition performance. The age when undergoing surgery and age at disease onset were positively associated with preoperative and chronic stage cognitive impairments; however, the age or disease duration did not show any effect on changes of cognitive impairments. HAMA and HDRS can measure the severity of anxiety and depression, respectively. However, disease duration was only found to be negatively associated with anxiety symptoms during the chronic stage (Table 3).

### 3.5 The effect of age and disease duration on the LEDD and programming setting

The stimulation parameters during the chronic stage were collected and used to calculate the TEED. However, there was no correlation between TEED and age or disease duration. Similarly, although STN‐DBS could significantly decrease the LEDD, as previously mentioned (Table 1), age and disease duration did not influence or change the LEDD during the preoperative and chronic stage.

## 4 DISCUSSION

Age and disease duration had a great impact on the symptoms and progression of PD; however, studies of these parameters did not obtain consistent results.^6^, ^7^, ^17^, ^18^ Therefore, we conducted the present study, which provided results concerning these issues based on our cohort of patients. To the best of our knowledge, this study was the first to investigate the influences of age and disease duration on the initial outcomes of STN‐DBS treatment of PD patients.

### 4.1 The age and disease duration influence the outcomes of DBS

The prevalence of PD increases with age, with a prevalence of 0.3% for those 55–64 years of age, 1.0% for those 65–74 years of age, 3.1% for those 75–84 years of age, and 4.3% for those 85–94 years of age,^4^ indicating that age plays an indispensable role in the pathophysiology of PD. Consistent with a previous study, our results indicated a negative correlation between age and medication response.^5^ Previously, patients older than 65 years were not traditionally considered as ideal candidates for STN‐DBS; however, the present study showed that ages ≥65 years should not be considered alone as exclusion criteria for STN‐DBS surgery because of the similar incidences of complications between patients of different ages,^19^ which extends the window of ages when undergoing surgery and raises the issue of whether there is an effect of age or disease duration on the efficacy of STN‐DBS.

Some previous studies have focused on the impact of age and disease duration on the outcomes of surgery. Rigidity scores were significantly decreased during the chronic stage of patients <70 years of age, but patients ≥70 years of age did not show similar results. Dyskinesia had a significantly greater improvement in patients ≥70 years of age when compared with patients <70 years of age during the chronic stage. A greater improvement in dyskinesia was found in patients with disease durations >10 years.^20^ However, Aygun et al.^6^ did not find any impact of age or disease duration on the efficacy of STN‐DBS. Another study conducted by Ory‐Magne et al.^7^ reported that age did not affect motor scores and PDQ‐39 improvements at 12 months. Nevertheless, at 24 months, there was a significant negative association between age and the improvement of three parameters of PDQ‐39 (mobility, activities of daily life, and cognition). Ten years after DBS surgery, the reduction of LEDD from baseline and dyskinesia scores were significantly lower in young‐onset PD patients. However, there was no difference in the voltage or volume of activated tissues of DBS settings between young‐onset and late‐onset patients.^21^ Dafsari et al.^18^ conducted PDQ‐8 domain analyses and reported that all parameters except cognition and emotional well‐being were significantly improved in patients aged <59 years, whereas only communication, activities of daily living, and stigma improved in patients aged 60–69 years, and activities of daily living and stigma in patients aged >70 years.

A correlation analysis between clinical evolution and age—rather than a comparison between two groups of PD patients arbitrarily split into a younger and an older group—was adopted in the present study, showing that correlation analysis was more discriminative if a continuous variable was predictive of the outcome.^7^ Some results were identical with those of other investigators; however, other results were not, which could be attributed to the different ranges of age and stages of surgery. The results of our analysis of chronic outcomes therefore suggested that late onset and elderly PD patients showed less therapeutic effects of STN‐DBS involving general, bradykinesia, and axial motor impairments; however, STN‐DBS may exert a better therapeutic effect on axial motor impairments of patients with longer disease durations. Regarding the initial outcomes of STN‐DBS, most results were similar with those of the chronic stage, showing that older patients could achieve a better amelioration of rigidity. Furthermore, we evaluated the changes in efficacy between initial and chronic stages. Early onset PD patients experienced a more obvious improvement in controlling general symptoms, especially rigidity and axial symptoms during the chronic stage, when compared with the initial stage. Notably, these improvements were also found in patients with longer disease durations. Overall, patients with an early time of surgery and disease onset, and long‐term disease duration may receive better motor improvements in general symptoms or some specific symptoms.

Health‐related quality could be divided into different classes. The results of our study on chronic outcomes indicated that late surgery time and disease onset patients may experience mild symptoms of a feeling of well‐being and stigma during the chronic stage. STN‐DBS may be specifically used to treat late onset patients who want to relieve stigma symptoms. This treatment may contribute to different social experiences at different ages. However, only a few results were found in cognition, anxiety, and depression observed using MoCA, HAMA, and HDRS. Similar with our results, previous studies also confirmed STN‐DBS relieved cognitive impairment during the first few years.^22^, ^23^ However, one group of PD patients with STN‐DBS showed that this therapeutic effect could disappear in 8 years,^23^ which may be related with disease progression. Early surgery and disease onset patients showed better cognitive performances during the preoperative and chronic stages of STN‐DBS, but age did not affect the relief of cognitive impairments. Patients with long‐term disease duration may have severe anxieties during the chronic stage, suggesting that these patients should be aware of anxiety and may need psychotherapy.

### 4.2 The potential mechanisms related with age and disease duration effects

There is no doubt that the age and disease duration had a great impact on the symptoms of PD and outcomes of STN‐DBS, so specific mechanisms may contribute to these observations. Morphology and independent component analysis identified PD‐specific atrophy in the midbrain, basal ganglia, basal forebrain, and medial temporal lobe, with an association between the degree of atrophy and clinical measures of disease severity.^24^ Yau et al. performed a longitudinal study of PD patients, which indicated that PD patients showed significantly greater cortical thinning than control subjects in the occipital and frontal lobes, and somatomotor‐sensory cortex. The atrophy patterns in the ventral frontal lobes resembled ones described in certain cases of Alzheimer's disease, which suggested disease spreading to the cortex may result in the onset of cognitive impairment.^25^

The morbidity of PD increases with aging, and both processes share similar cellular and other alterations in the dopaminergic pathways and other systems.^26^, ^27^ Healthy aging is accompanied by loss of dopaminergic neurons,^28^ and it is assumed that the symptoms of PD appear when dopaminergic neurons in the substantia nigra are lost by up to 60%–70%.^29^ A previous study found the right dorsal premotor area, precentral, parietal lobule, and bilateral precuneus were more activated in PD patients, when compared with old control subjects, when imagining stepping over obstacles. In a similar manner, the premotor area, precentral and parietal lobes, and visual association areas were activated in older subjects, when compared with younger subjects.^30^

Another study investigated electroencephalographic changes during an auditory oddball task while walking in young adults, older adults, and patients with PD, and found that old adults and patients with PD showed prolonged P300 latency, when compared with young adults. Furthermore, the authors found that better motor and cognitive performances were correlated with shorter P300 latencies.^31^ Aging may therefore be an extra border to PD pathology. Phase‐amplitude coupling (PAC) between beta and gamma oscillations as well as beta burst features can be identified as electrophysiological biomarkers for PD, and an additional study confirmed that PAC between beta and gamma activity was elevated in elderly subjects, when compared to younger subjects without PD.^26^ Elevated beta oscillatory activity in the STN is considered as the most pronounced abnormal electrophysiological signature of PD,^32^ with a correlation found between beta power and bradykinesia.^33^ However, it could be relieved by levodopa and DBS^34^, ^35^; moreover, an incidence of longer beta bursts in the STN has been confirmed to be positively correlated with clinical impairments.^36^ These findings may illustrate the reasons why aging and disease duration showed a great impact on the outcomes of STN‐DBS in PD patients; nevertheless, future studies investigating the effects of age and disease duration on the electrophysiological signatures of STN may provide better answers.

The previous study measured cerebral 5‐HT_1B_R binding in a group of PD patients with STN‐DBS, and found that they exhibited a significant loss of frontal and parietal 5‐HT_1B_R, which indicated STN‐DBS dynamically regulated the serotonin system in PD,^37^ but whether this regulation was affected by age and disease duration still needs to be investigated. Hahn et al. found monetary gain‐induced stronger increases in ventral striatum dopamine synthesis than that of the loss in young men; however, the opposite effect was discovered in young women, indicating a neurobiological basis for known behavioral sex differences in reward and punishment processing.^38^ Chandra et al. revealed major sex disparities (young male and female rats) in gene expression and canonical pathways of microvessels and these differences provided a foundation to study neurological diseases.^39^ Meanwhile, a previous study showed that young women had significantly higher cerebral blood flow than men in the frontal and temporal lobes, but these differences disappeared at age 65 years.^17^ PD mainly affects elderly people and the sample size in our study was limited to investigate the effect of different sexes on the PD symptoms.

Our study emphasized and illustrated the effect of age and disease duration on the outcomes of STN‐DBS; however, multicenter studies with a larger cohort of patients should be conducted in the future to strengthen and confirm our observations.

## 5 CONCLUSION

The present study investigated the effects of age and disease duration on initial and chronic efficacies of STN‐DBS. During the initial stage, the age when undergoing surgery and disease onset were negatively correlated with the effects on bradykinesia, with a better control of general symptoms in long‐term disease patients. In a similar manner, patients with an early time of surgery and disease onset, and long‐term disease duration showed better control of bradykinesia and axial symptoms at the chronic stage. Furthermore, long‐term disease duration and early disease onset increased the therapeutic efficacy of general, rigid, and axial symptoms with STN‐DBS after a long period. Nevertheless, patients with late disease onset achieved a better relief of stigma.

## AUTHOR CONTRIBUTION

Tingting Du, Yingchuan Chen, Guanyu Zhu, Tianshuo Yuan, Xin Zhang and Ruoyu Ma were involved in data collection. Tingting Du, Yingchuan Chen, Guanyu Zhu, and Jianguo Zhang were involved in data analysis. Jianguo Zhang conducted the surgery and designed the study. Tingting Du and Yingchuan Chen wrote the article.

## CONFLICT OF INTEREST

None declared.

## Data Availability

The data that support the findings of this study are available from the corresponding author upon reasonable request.
